# Diagnosis and embolization of a non-iatrogenic and non-traumatic thyrocervical pseudoaneurysm

**DOI:** 10.1186/s42155-026-00675-x

**Published:** 2026-04-16

**Authors:** Qian Yang, Amit Ramjit

**Affiliations:** Department of Radiology, Maimonides Medical Center, 4802 10 Avenue, Brooklyn, NY 11219 USA

**Keywords:** Thyrocervical trunk, Pseudoaneurysm, N-butyl cyanoacrylate, Endovascular embolization, Interventional radiology, Case report

## Abstract

Non-traumatic and non-iatrogenic pseudoaneurysms of the thyrocervical trunk are exceedingly rare. We report a case of a 63-year-old man with esophageal cancer who presented with fever and worsening chronic cough following initiation of induction chemotherapy. Computed tomographic angiography demonstrated a 1.3 cm pseudoaneurysm arising from the thyrocervical trunk. The lesion was successfully occluded by selective endovascular embolization using N-butyl cyanoacrylate (NBCA) liquid embolic, achieving complete occlusion while preserving flow to adjacent branches. The patient recovered uneventfully, and follow-up imaging demonstrated sustained occlusion of the pseudoaneurysm. This case highlights the safety and efficacy of endovascular embolization as a minimally invasive alternative to open surgery for the management of thyrocervical trunk pseudoaneurysms.

## Introduction

Pseudoaneurysm of the thyrocervical trunk and its branches is a rare vascular condition. The most common etiologies are iatrogenic injuries, typically associated with central venous cannulation or arterial catheterization [1–3]. Traumatic causes, typically involving penetrating injuries, have also been reported but are less common [4]. In contrast, non-iatrogenic and non-traumatic cases are exceedingly rare, with only one idiopathic instance documented in the literature [5]. In this case report, we present a rare, non-iatrogenic, non-traumatic pseudoaneurysm arising from a branch of the thyrocervical trunk in a patient with esophageal carcinoma with pulmonary invasion, successfully managed with transcatheter embolization using glue.

## Case report

A 63-year-old male with no traumatic history and a recent diagnosis of esophageal cancer presented to the emergency room for fever and worsening chronic cough after onset of induction chemotherapy. The patient is a non-surgical candidate who has never had operative or procedural intervention of the cancer except for a diagnostic esophagogastroduodenoscopy. On the chest computed tomography (CT), the patient was found to have a large cavitary mass in the apex of the right lung and was admitted for treatment of septic shock and pneumonia. During the admission, the patient developed worsening hoarseness. Esophagram confirmed a fistula from the proximal esophagus to the right upper lobe. An esophageal stent was placed by gastroenterology. Despite the treatment, the patient developed hemoptysis and acute respiratory failure requiring high flow oxygen. Chest computed tomography angiography (CTA) was obtained, showing a hyperdensity measuring 1.3 cm at the medial aspect of the right upper lobe mass, concerning a pseudoaneurysm possibly arising from bronchial or pulmonary artery (Fig. 3A). Given the absence of prior imaging for comparison and potentially unfavorable outcome of rupture, interventional radiology was consulted for diagnostic transcatheter angiography and possible embolization.

**Fig. 1:**
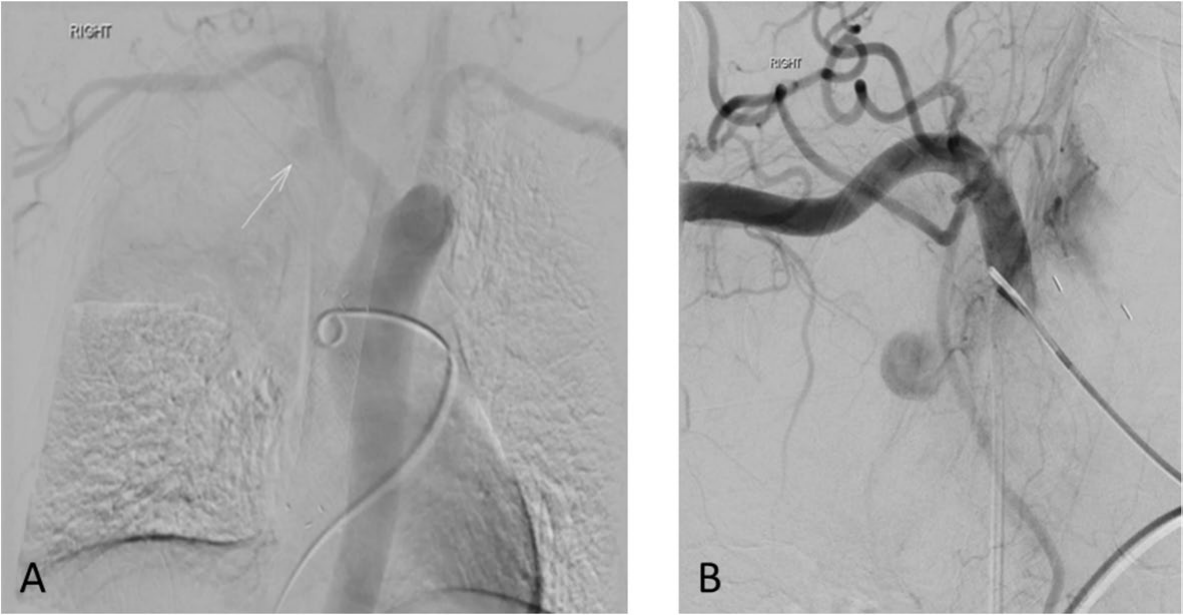
**A** Right pulmonary artery angiography showing a round density (arrow) adjacent to the brachiocephalic artery during systemic arterial filling. **B** Selective angiography of the brachiocephalic artery confirmed the presence of a pseudoaneurysm

**Fig. 2:**
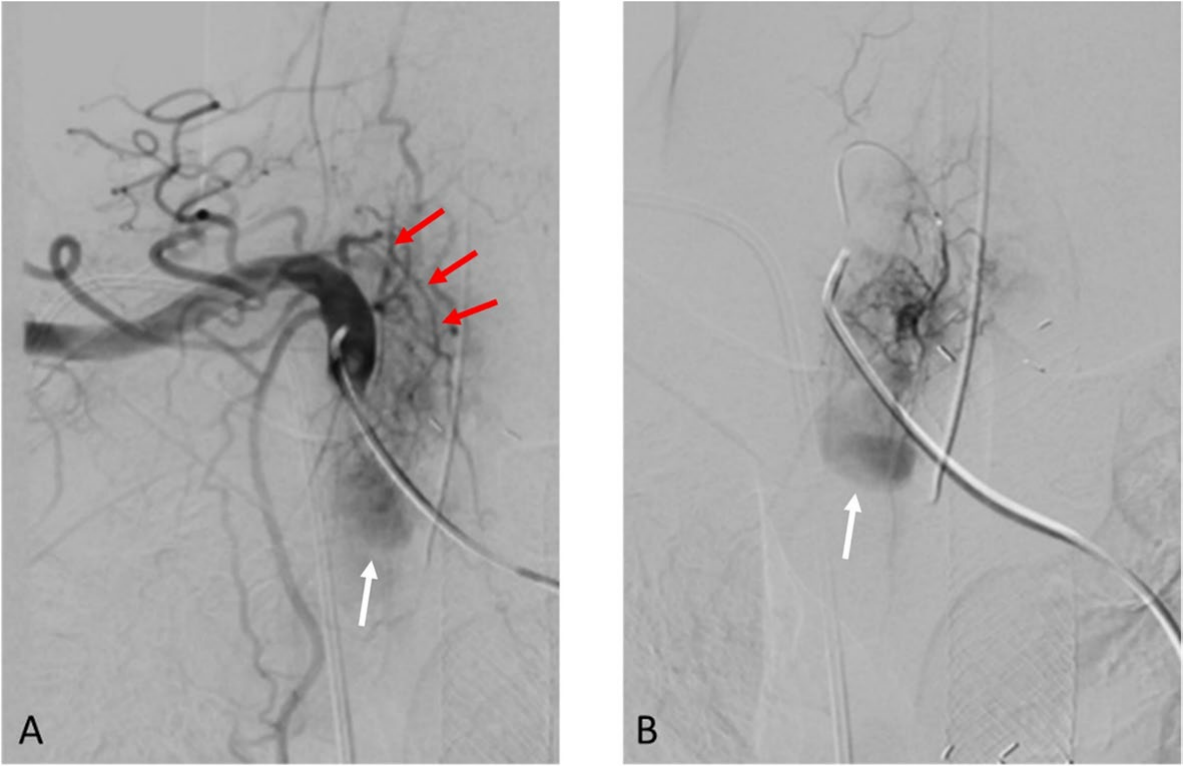
**A** Selective angiography of the brachiocephalic artery showing the pseudoaneurysm (white arrow) with the feeding artery (red arrows). **B** Superselective angiography of an ascending branch of the brachiocephalic/subclavian artery showing opacification of a pseudoaneurysm (white arrow), most likely arising from the suprascapular branch of the thyrocervical trunk

**Fig. 3:**
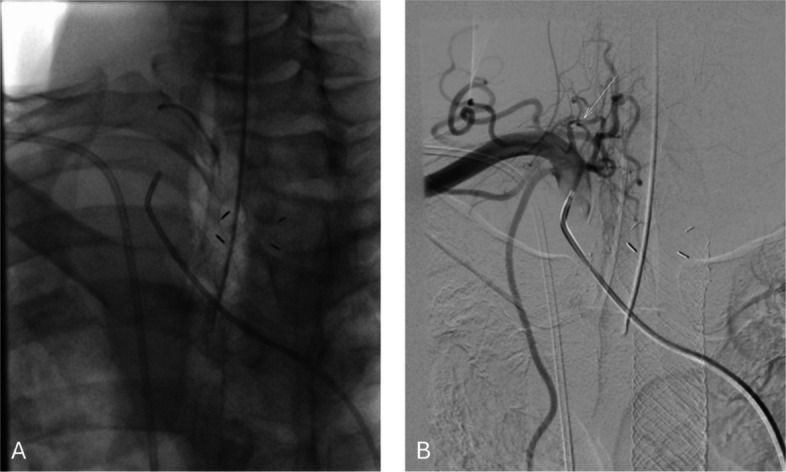
**A** Glue cast following embolization, demonstrating radiopaque accumulation of NBCA within the proximal segment of the feeding artery. **B** Post-embolization angiogram showing no opacification of the pseudoaneurysm confirming complete occlusion, with the glue cast visualized at the embolization site (arrow)

During the procedure, multiple digital subtraction angiographies (DSA) were performed. The right common femoral (RCF) arterial access was initially obtained using a 5 Fr 10 cm sheath. Thoracic aorta was catheterized using 5 Fr Sos and Mikaelsson Kumpe catheters, and 0.035 Bentson wire with angiography showed no active hemorrhage or pseudoaneurysm coming from the bronchial arteries. The RCF venous access was obtained using a 6 Fr 10 cm sheath. The pulmonary arterial system was catheterized using a 6 Fr angled pigtail catheter and 0.035 Bentson wire with the right pulmonary artery angiography showing no evidence of pseudoaneurysm from the pulmonary arteries but a round density adjacent to the brachiocephalic artery during the systemic arterial filling (Fig. 1A). The catheter and wire were then redirected through the RCF arterial access with a selective angiography of the brachiocephalic artery performed, confirming the presence of a pseudoaneurysm with no active extravasation (Fig. 1B). Superselective angiography using 2.8 Fr and 2.4 Fr Progreat microcatheters and 0.018 Fathom microwire of the right internal thoracic artery showed no opacification of the pseudoaneurysm. Unfortunately, the patient then developed excessive coughing and further explorative angiographies were aborted due to excessive motion. Three days later, a second catheterized angiography was scheduled with general anesthesia. During the procedure, the brachiocephalic artery was catheterized using a 6 Fr Penumbra select catheter and 0.035 Bentson wire and an ascending branch arising at the brachiocephalic-subclavian junction was superselected using a 2.0 Fr TruSelect microcatheter and 0.016 Syncro microwire, confirming the presence of the pseudoaneurysm that most likely arise from a branch of the thyrocervical trunk (Fig. 2). Based on the predominantly inferior course of this vessel, the involved branch was felt to most likely represent the suprascapular artery; however, its relatively medial orientation compared with typical anatomy may reflect reported anatomic variability and could have been further influenced by tumor-related vascular recruitment and distortion [6]. Pre-embolization, selective angiography of the thyrocervical trunk and adjacent arterial branches demonstrated no evidence of dual arterial supply or collateral flow. Given the distal location and presumed narrow neck of the pseudoaneurysm, embolization was performed using N-butyl cyanoacrylate (NBCA) liquid embolic in a 1:6 ratio with post-embolization DSA demonstrating no filling of pseudoaneurysm (Fig. 3). Post-procedure, the patient reported significant improvement in coughing and hemoptysis and was discharged home in a few days.

**Fig. 4:**
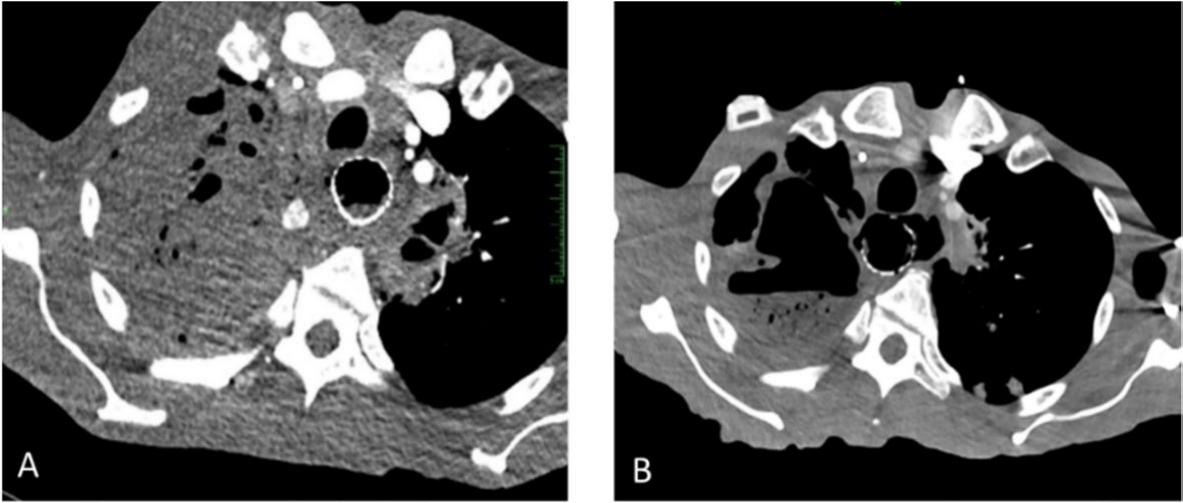
**A** Initial chest CTA during the first admission showing a hyperdensity measuring 1.3 cm at the medial aspect of the right upper lobe mass. **B** Post intervention chest CTA during the second admission showing no evidence of pseudoaneurysm

The patient resumed chemotherapy after discharge but unfortunately developed worsening symptoms a few months later. During the second admission, a second CTA of the chest showed persistent right upper lobe cavitary mass with progressed fistulation of the esophagus involving the bilateral upper lobes but with no evidence of pseudoaneurysm (Fig. 4). Despite treatment with antibiotics and vasopressors, the patient continued to decompensate and died 4 days after admission.

## Discussion

The thyrocervical trunk is the second ascending branch of the subclavian artery and is located in zone I of the neck. Pseudoaneurysms of peripheral arteries in the upper extremity are rare, with thyrocervical trunk pseudoaneurysms being the second most common [7]. Most reported cases are iatrogenic, typically resulting from vascular injury following central venous or hemodialysis catheter placement [2, 3]. Non-iatrogenic causes are less common due to the vessels’ deep location and are usually associated with penetrating trauma [2]. There has only been one case of idiopathic thyrocervical pseudoaneurysm reported that involves neither iatrogenic nor traumatic injuries [5]. While small pseudoaneurysms may remain asymptomatic for years, larger lesions often present as painless, pulsatile masses that can compress surrounding structures. Treatment is generally recommended due to potential complications such as pain, mass effect, and notably rupture with associated high mortality risk [7].

**Fig. 5:**
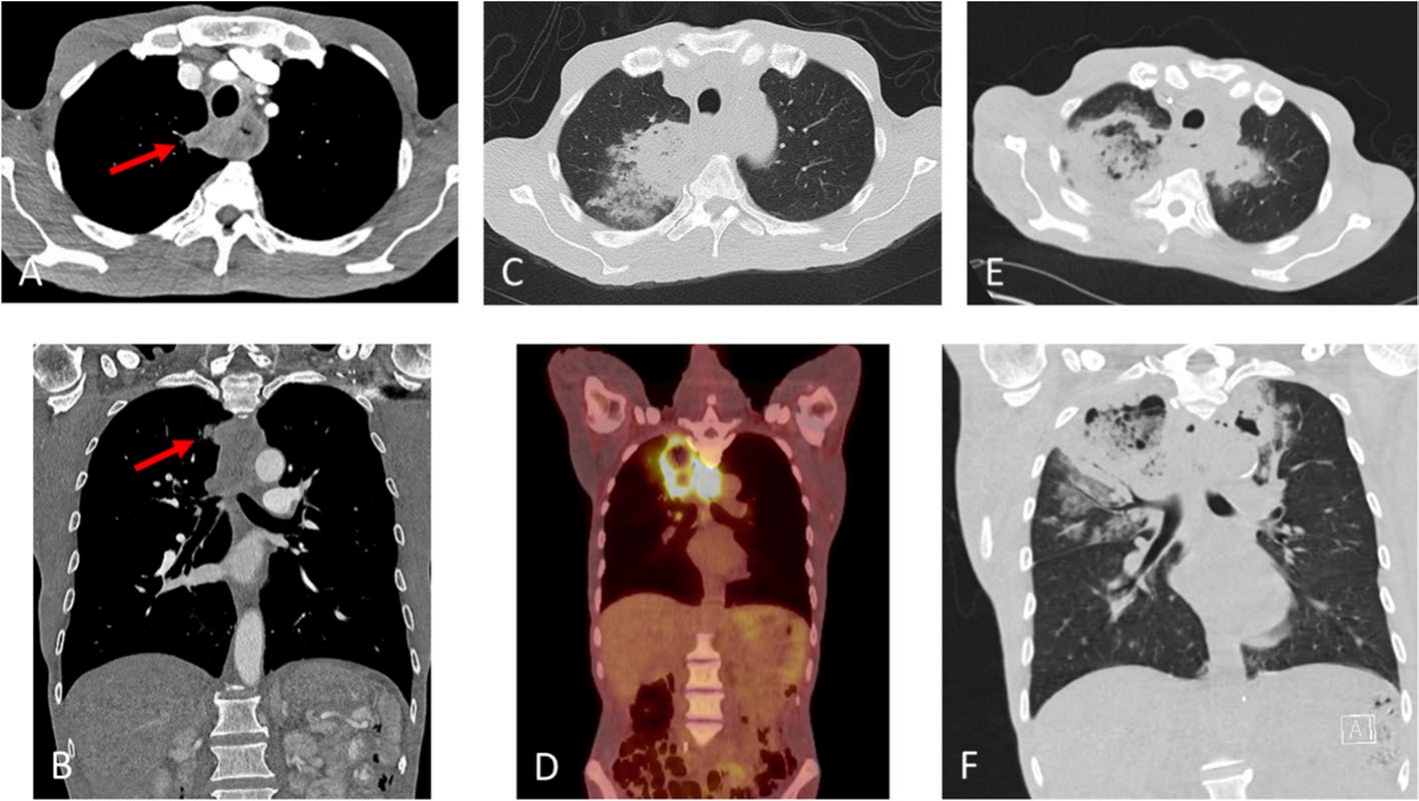
**A**, **B** Initial chest CT showing a soft tissue mass in the proximal esophagus with small nodular invasion to the right upper lobe. **C**, **D** PET/CT 3 weeks later showing a large necrotic mass in the right upper lobe concerning for an extending abscess from the adjacent esophageal lesion. **E**, **F** Chest CT at the emergency room 1 week after induction chemotherapy and 3 months after initial imaging showing an expanding right upper lobe cavitary mass

There is extremely limited documentation of non-iatrogenic and non-traumatic thyrocervical pseudoaneurysm with only one case reported up to date where no known cause of the pseudoaneurysm was identified [7]. In our case, the patient has a known history of esophageal cancer with quick and aggressive invasion into the right upper lobe of the lung, where many branches of the brachiocephalic and subclavian arteries reside, including the thyrocervical trunk (Fig. 5). In addition, this patient had superimposed infection at the site of the cavitary lung metastasis likely due to persistent aspiration pneumonia secondary to the esophageal-pulmonary fistula that was confirmed on the esophagram. While there is no well-documented clinical explanation for the development of non-iatrogenic, non-traumatic thyrocervical trunk pseudoaneurysms, case reports have described pulmonary artery pseudoaneurysms in the context of regional lung cancer treated with systemic chemotherapy and/or radiotherapy [8–12]. Additionally, chronic infection related to mediastinitis following invasive thymoma resection and postoperative irradiation has been associated with the formation of brachiocephalic pseudoaneurysms [9]. Taken together, we speculate that the pseudoaneurysm of the thyrocervical trunk in our patient may be attributable to a combination of factors, including expanding tumor necrosis, vascular wall erosion due to direct tumor invasion or concurrent infection, and potential vascular injury following chemotherapy.

**Fig. 6:**
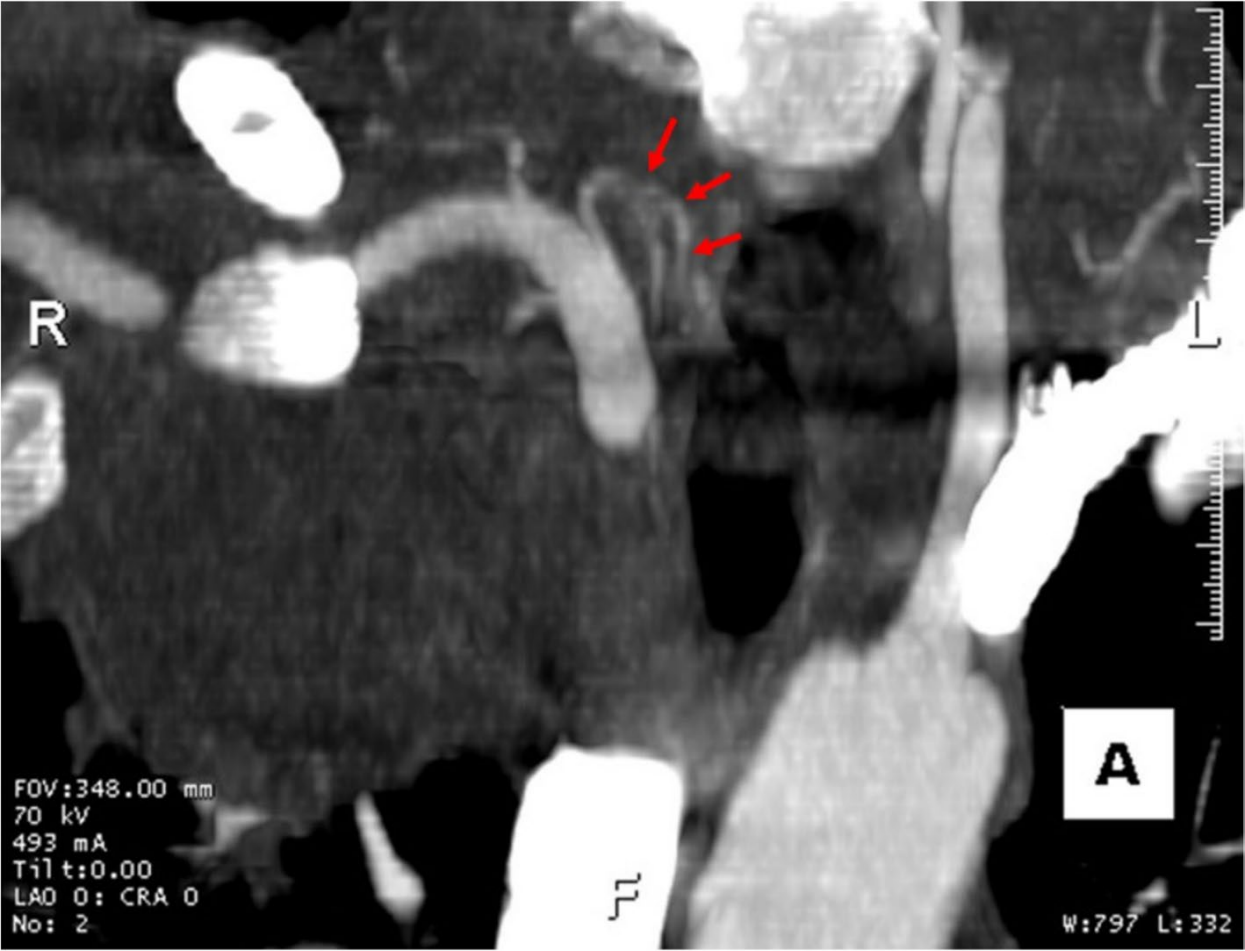
Preprocedural CTA MIP image demonstrating the proximal takeoff of an arterial branch arising near the brachiocephalic–subclavian junction (red arrows). No definite arterial connection to the pseudoaneurysm was identified on prospective review. Following catheter-directed angiography, retrospective correlation demonstrated that this vessel corresponded to the branch confirmed to supply the pseudoaneurysm

While both CTA and duplex sonography are reliable tools for evaluating suspected pseudoaneurysms, catheter-directed DSA remains the gold standard for its superior delineation of collateral vasculature [5]. In our case, the hyperdensity adjacent to the brachiocephalic artery seen on the initial chest CTA raised suspicion for a pseudoaneurysm, possibly originating from the pulmonary or bronchial arteries—rare entities, but more frequently reported than thyrocervical pseudoaneurysms, especially in non-iatrogenic settings. These are thought to result from increased shear stress on a weakened arterial wall, which may occur in the context of bronchopulmonary infections or chronic conditions such as hypertension, tachycardia, and obstructive lung disease [13]. Prospective review of the pre-procedural CTA showed no definitive arterial feeder to the pseudoaneurysm as only the proximal origin of a suspected branch could be visualized with limited delineation of its distal course. However, catheter-directed angiography subsequently identified the responsible vessel and confirmed the much rarer thyrocervical origin, allowing retrospective CT correlation along a similar trajectory (Fig. 6). This finding underscores the superior diagnostic utility of catheter-directed DSA and highlights the importance of thorough evaluation in regions with complex vascular anatomy, such as the lung apices.

Pseudoaneurysms of the thyrocervical trunk can be managed through various approaches. Classic management through open surgical repair is associated with significant morbidity and mortality, longer recovery, and higher costs [10]. While ultrasound-guided interventions such as compression repair or thrombin injection provide a safer and more economical alternative with a high success rate, its utility is limited to superficial pseudoaneurysms. Advancements in endovascular techniques have emerged as a safe alternative, offering shorter recovery times and allowing the management of deeper and more complex lesions [1, 3]. Most of the reported endovascular interventions involve the use of coils, which are historically favored for their precise deployment and controlled occlusion, with a minority of cases using adhesive or non-adhesive liquid embolics such as NBCA or ethylene vinyl alcohol (EVOH) as a complemental embolic agent. Although less common, successful embolization of thyrocervical trunk pseudoaneurysms using liquid embolics alone has been reported, following DSA confirmation of no collateral connections to adjacent subclavian artery branches [14, 15]. Consistent with previous case reports, our case supports the use of liquid embolics alone as a safe, efficient, and cost-effective option for embolization of thyrocervical trunk pseudoaneurysms in selective cases.

Non-iatrogenic, non-traumatic pseudoaneurysm of the thyrocervical trunk is an exceedingly rare condition, with only two cases reported in the literature, including our own. While the exact mechanism is unclear, we propose that a combination of factors—such as direct tumor invasion and post-therapy vascular injury—may contribute to pseudoaneurysm formation in the setting of tumor necrosis and infection. Catheter-directed digital subtraction angiography is the diagnostic gold standard, and thorough evaluation is critical for precise localization in anatomically complex regions. Finally, in select cases, liquid embolics may offer a safe, effective, and cost-efficient option for endovascular management in selected cases.

## Data Availability

Not applicable.
